# Extracellular IL-37 promotes osteogenic differentiation of human bone marrow mesenchymal stem cells via activation of the PI3K/AKT signaling pathway

**DOI:** 10.1038/s41419-019-1904-7

**Published:** 2019-10-03

**Authors:** Chenyi Ye, Wei Zhang, Kai Hang, Mo Chen, Weiduo Hou, Jianzhong Chen, Xi Chen, Erman Chen, Lan Tang, Jinwei Lu, Qianhai Ding, Guangyao Jiang, Baojian Hong, Rongxin He

**Affiliations:** 1 Department of Orthopedic Surgery, the Second Affiliated Hospital, School of Medicine, Zhejiang University, No. 88, Jiefang Road, 310009 Hangzhou, China; 2 Orthopedics Research Institute of Zhejiang University, No. 88, Jiefang Road, 310009 Hangzhou, China; 3 Department of Rheumatology, Second Affiliated Hospital, School of Medicine, Zhejiang University, Hangzhou, China; 4 Institute of Immunology, School of Basic Medical Sciences, Zhejiang University, No. 866, Yuhangtang Road, 310000 Hangzhou, China; 5 Department of Epidemiology & Health Statistics, School of Public Health, School of Medicine, Zhejiang University, 310058 Hangzhou, China; 6 Department of Central Laboratory Medicine, Zhejiang Provincial People’s Hospital, Hangzhou, China; 7 Department of Central Laboratory Medicine, People’s Hospital of Hangzhou Medical College, Hangzhou, China

**Keywords:** Differentiation, Mesenchymal stem cells

## Abstract

Interleukin (IL)-37, a pivotal anti-inflammatory cytokine and a fundamental inhibitor of innate immunity, has recently been shown to be abnormally expressed in several autoimmune-related orthopedic diseases, including rheumatoid arthritis, ankylosing spondylitis, and osteoporosis. However, the role of IL-37 during osteogenic differentiation of mesenchymal stem cells (MSCs) remains largely unknown. In this study, extracellular IL-37 significantly increased osteoblast-specific gene expression, the number of mineral deposits, and alkaline phosphatase activity of MSCs. Moreover, a signaling pathway was activated in the presence of IL-37. The enhanced osteogenic differentiation of MSCs due to supplementation of IL-37 was partially rescued by the presence of a PI3K/AKT signaling inhibitor. Using a rat calvarial bone defect model, IL-37 significantly improved bone healing. Collectively, these findings indicate that extracellular IL-37 enhanced osteogenesis of MSCs, at least in part by activation of the PI3K/AKT signaling pathway.

## Introduction

Large bone defects or non-unions are one of the most common complications following severe fracture, bone tumor ablation, and debridement of a wide range of bone infections and congenital defects; these complications continue to be a challenge for orthopedic surgeons. Despite significant developments in surgical treatments, including autologous and allologous bone grafting, the obvious drawbacks, including donor shortage, donor-site morbidity, infection, and immune rejection, still remain unresolved, which has largely limited their clinical application^1,2^. To circumvent these limitations, bone tissue engineering approaches using cell seeding, scaffold applications, and cytokine activation have provided novel treatment options for large bone defects or non-unions^3^.

As a major contributor to bone formation, mesenchymal stem cells (MSCs) retain self-renewal capability and multi-lineage differentiation capacity into various mesodermal tissues including bone and cartilage, and they have been reported to play a key role in the healing of bone defects. Among the various osteogenic seed cells, bone marrow MSCs (BMSCs) are easy to harvest from abundant sources and exhibit reduced donor morbidity, making them a promising candidate for massive bone repair^4–6^. Moreover, the administration of BMSCs has been applied for the treatment of several diseases, including bone defects, rheumatoid arthritis (RA), and osteoarthritis, with promising results not only in animal models but also in clinical trials as well^7–9^. Although the osteogenic potential of BMSCs during bone healing remains poorly understood, recent studies have suggested that bone healing is not induced by seeded BMSCs alone but is a result of interactions with host cells and a variety of cytokines that they release^10,11^.

Mounting evidence has revealed a connection between bone metabolism and immune-mediated inflammatory diseases^12,13^. According to an epidemiological survey, RA induces low bone turnover, resulting in a twofold increase in the incidence of osteoporosis (OP) and a 1.35–2.13-fold increase in the fragility fracture risk^12,14^. A variety of inflammatory cytokines were recently found to affect the osteogenic differentiation of stem cells^15–18^. Yang et al. reported that interleukin (IL)-8 enhanced the therapeutic effects of BMSCs in bone regeneration via the phosphoinositide-3 kinase (PI3K)/AKT signaling pathway^16^. Huh et al. reported that IL-6 enhanced osteogenic differentiation of stem cells by activating signal transducer and activator of transcription factor 3^15^. In an in vitro study by Kukolj et al., IL‐33 was found to guide osteogenesis in dental stem cells^17^. On the other hand, another important inflammatory cytokine, IL-1β, was found to suppress osteogenesis and adipogenesis of MSCs^18^. Considering the inflammatory, ischemic environment of large bone defects and non-union sites, we believe that further in vitro and in vivo studies focusing on the effects of inflammatory cytokines are urgently needed to uncover the underlying molecular mechanism of bone regeneration and aid in the development of novel effective methods to accelerate bone healing.

IL-37 [formerly IL-1 family member 7], a newly identified member of the IL-1 family, is produced as a precursor protein that is processed by caspase-1, releasing the mature form of IL-37^19^. IL-37 includes five splice variants (IL-37a–e) and acts as a cytokine with both intracellular and extracellular functionality^20^. Recently, studies have observed abnormal expression of IL-37 in several autoimmune-related orthopedic diseases, such as RA and ankylosing spondylitis (AS)^21^. Chen et al. demonstrated a connection between IL-37 and several bone metabolism-related inflammatory cytokines and reported that recombinant IL-37 inhibited the expression of pro-inflammatory cytokines, including tumor necrosis factor (TNF)-α, IL-6, IL-17, and IL-23, in peripheral blood mononuclear cells (PBMCs) in patients with AS^22^. Intriguingly, serum levels of IL-37 were positively associated with the activity and severity of RA^21,23^. Moreover, Fawzy et al. reported that the serum level of IL-37 was significantly elevated in patients with OP^24^. Jafari et al. demonstrated that IL-37 inhibits osteoclast formation and bone resorption in vivo^25^, again suggesting the regulatory effect of IL-37 on bone metabolism and osteogenesis. However, to date, the function and regulation of IL-37 in MSCs have not been reported.

Herein, we investigate the effects of IL-37 on osteogenic differentiation of MSCs. By assessing the expression levels of specific markers and mineral deposition, we revealed that IL-37 promoted osteogenic differentiation of human BMSCs partly via activation of the PI3K/AKT signaling pathway in vitro. Moreover, using a rat calvarial defect model, we showed that IL-37 improved bone healing in vivo.

## Results

### IL-37 had no cytotoxicity on BMSC proliferation

**Fig. 1:**
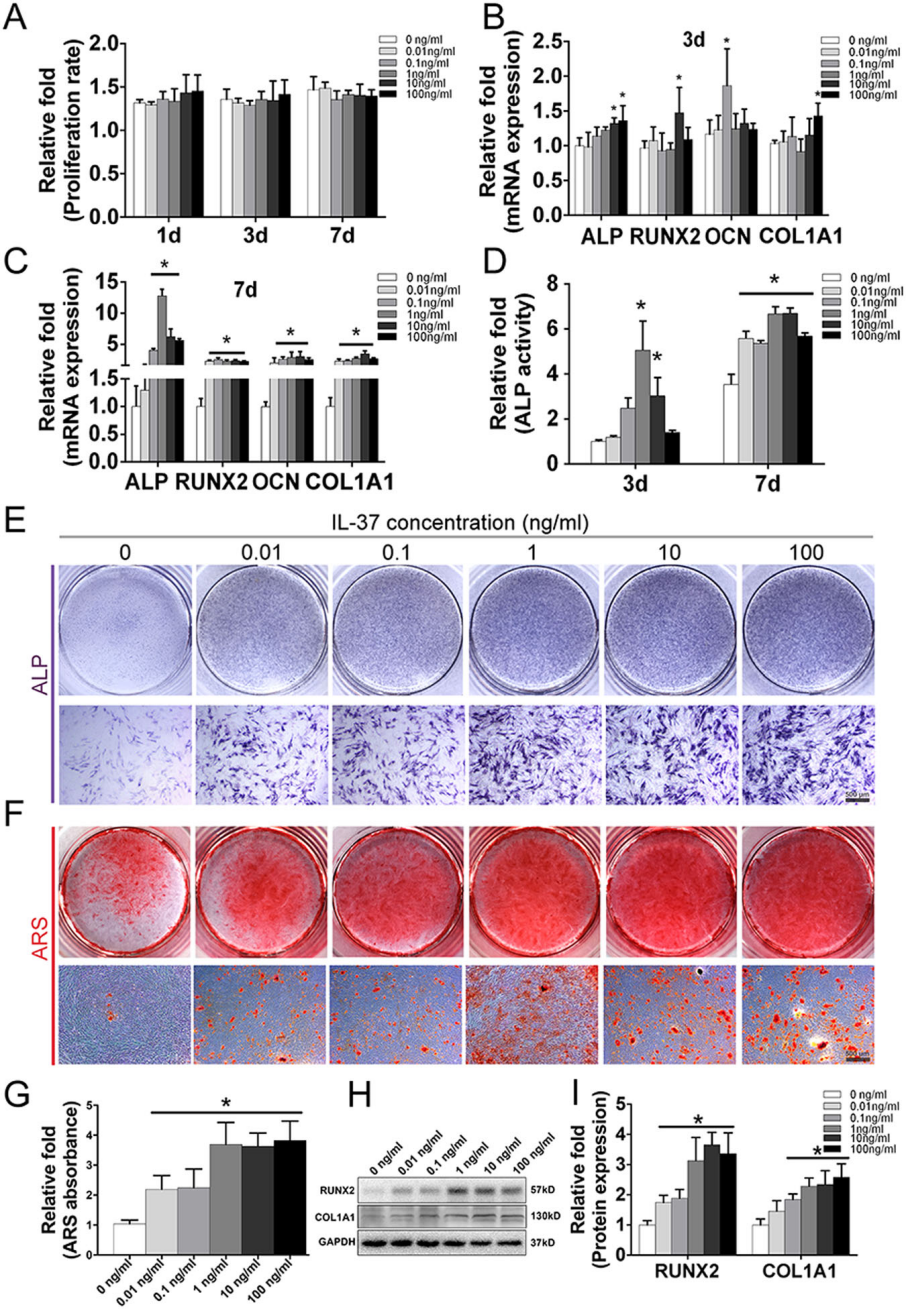
Effects of IL-37 on osteogenic differentiation of BMSCs. **a** The effects of IL-37 on BMSC proliferation on days 1, 3, and 7. **b** Relative mRNA expression of osteo-specific genes (ALP, RUNX2, COL1A1, and OCN) on day 3 of osteogenesis; **c** Relative mRNA expression of osteo-specific genes (ALP, RUNX2, COL1A1, and OCN) on day 7 of osteogenesis; **d** Relative ALP activity on days 3 and 7 of osteogenesis; **e** Results of ALP staining on day 7 of osteogenesis; **f** Results of ARS on day 14 of osteogenic differentiation; **g** Relative quantitative analysis of the ARS; **h** Western blot analyses of osteo-specific proteins including RUNX2 and COL1A1 on day 7 of osteogenesis; **i** Relative quantitative analysis of western blot analyses for RUNX2 and COL1A1. OCN osteocalcin, ALP alkaline phosphatase, ARS alizarin red staining. Scale bars = 500 μm. *P < 0.05 vs. control group

To determine whether IL-37 influence the viability of human BMSCs, Cell Counting Kit-8 (CCK-8) analysis was performed. The effects of IL-37 on BMSC proliferation on days 1, 3, and 7 are shown in Fig. 1a. The results showed no decrease in the relative proliferation rate of BMSCs. In detail, although the proliferation rate was increased when treated with IL-37 at concentrations between 10 and 100 ng/ml on days 1 and 3, no significant difference was detected between 0.01 and 100 ng/ml on day 7.

### IL-37 increased the levels of osteo-specific genes

To assess the role of IL-37 in the osteogenic differentiation of BMSCs, the levels of osteo-specific genes, including alkaline phosphatase (ALP), RUNX2, COL1A1, and osteocalcin (OCN) were detected by real-time polymerase chain reaction (RT-PCR) on days 3 and 7. The results of RT-PCR at day 3 revealed an increase of ALP, RUNX2, COL1A1, and OCN mRNA levels when BMSCs were treated with recombinant human IL-37 (rhIL-37) at certain concentrations (Fig. 1b). On day 7 of osteogenesis, significantly increased mRNA levels of ALP, RUNX2, COL1A1, and OCN were observed in all rhIL-37-treated groups, compared with the control group (*P* < 0.05; Fig. 1c).

### IL-37 promoted ALP activity and calcium deposit formation

ALP activity, an important marker of early-stage osteogenesis, was detected using both an ALP Activity Assay Kit and ALP staining. Compared with the control group, greater ALP activity was observed in the 1 and 10 ng/ml IL-37 groups on day 3, whereas the IL-37 with concentrations range from 0.01 to 100 ng/ml significantly improved the ALP activity on day 7 (Fig. 1d). Similar results were observed with ALP staining on day 7 (Fig. 1e). Calcium deposits were examined by alizarin red staining (ARS) and quantified on day 14. Being consistent with the results of ALP staining, more calcium deposits were observed in the IL-37-treated groups with concentrations range from 0.01 to 100 ng/ml (Fig. 1f, g).

### IL-37 increased the levels of osteo-specific proteins

**Fig. 2:**
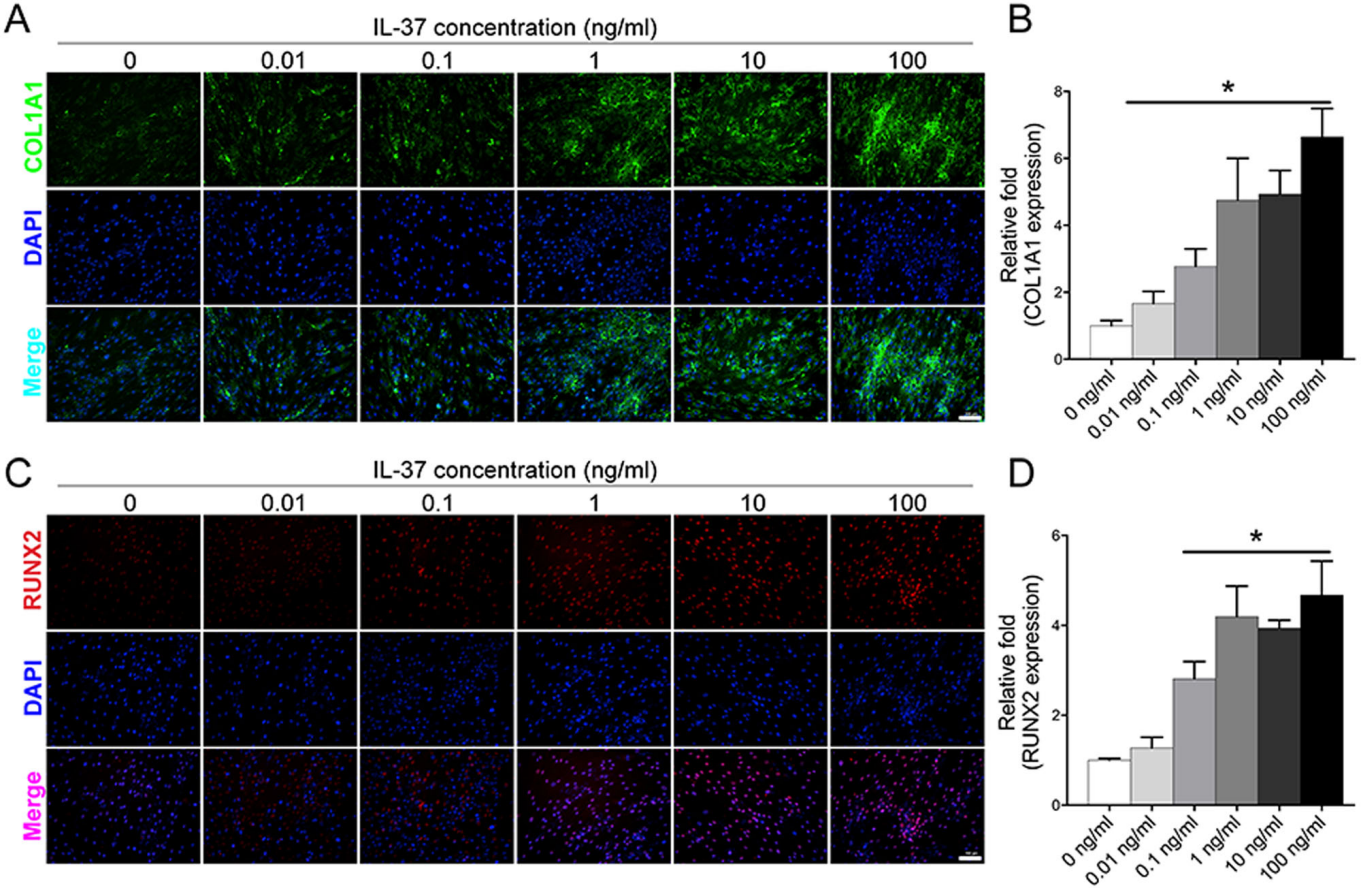
Immunofluorescence analyses showed that IL-37 increased the protein expression of RUNX2 and COL1A1. **a** IL-37 increased the protein expression of RUNX2 on day 3 after the induction of osteogenesis; **b** Relative quantitative analysis of RUNX2 immunofluorescence staining; **c** IL-37 increased the protein expression of COL1A1; **d** Relative quantitative analysis of COL1A1 immunofluorescence staining. Scale bars = 100 μm.*P < 0.05 vs. control group

Western blot analysis on day 7 showed that IL-37 (0.01–100 ng/ml) significantly increased the protein expression of RUNX2, whereas the COL1A1 protein level was increased when treated with IL-37 at a concentration of 0.1–100 ng/ml (Fig. 1h, i). In addition, we also used immunofluorescence (IF) to confirm the expression of RUNX2 and COL1A1 proteins, which revealed that IL-37 at a concentration of 0.1–100 ng/ml increased the protein levels of both RUNX2 and COL1A1 on day 3 (Fig. 2a–d).

### The PI3K/AKT signaling pathway was activated due to the presence of IL-37

**Fig. 3:**
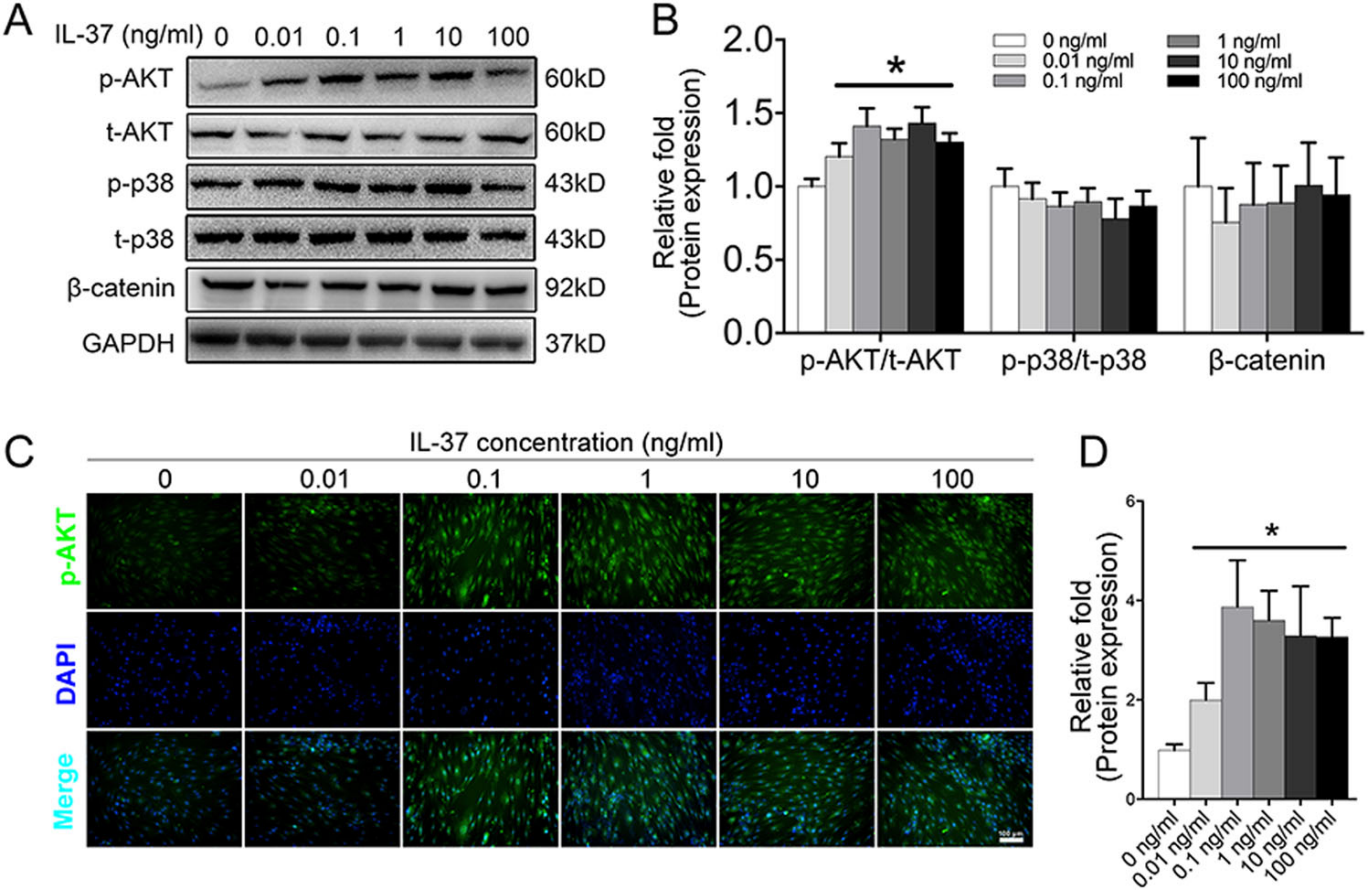
IL-37 activated the PI3K/AKT signaling pathway. **a** Western blot analyses of specific proteins in β-catenin, MAPK/p38, and PI3K/AKT pathways on day 3 of osteogenesis; **b** Relative quantitative analysis of western blot analyses. **c** Immunofluorescence staining showed that IL-37 increased the protein expression of p-AKT; **d** Relative quantitative analysis of p-AKT immunofluorescence staining. Scale bars = 100 μm.*P < 0.05 vs. control group

To explore the underlying signaling pathways involved in the regulation of BMSC differentiation by IL-37, several most common signaling pathways involved in osteogenesis, including the β-catenin, mitogen-activated protein kinase (MAPK)/p38, and PI3K/AKT signaling pathways, were examined by western blotting. Increased expression of p-AKT was observed in the IL-37 group (0.01–100 ng/ml) on day 3^26–29^ of osteogenic differentiation, whereas no significant differences were found in the protein levels of t-AKT, p-p38, t-p38, and β-catenin among these groups (Fig. 3a, b). Moreover, IF analysis confirmed increased expression of p-AKT accumulation in the IL-37 group (0.01–100 ng/ml) compared with the control group (Fig. 3c, d).

### The enhanced osteogenic differentiation of MSCs due to the supplement of IL-37 was partially rescued by the presence of PI3K/AKT signaling inhibitor

**Fig. 4:**
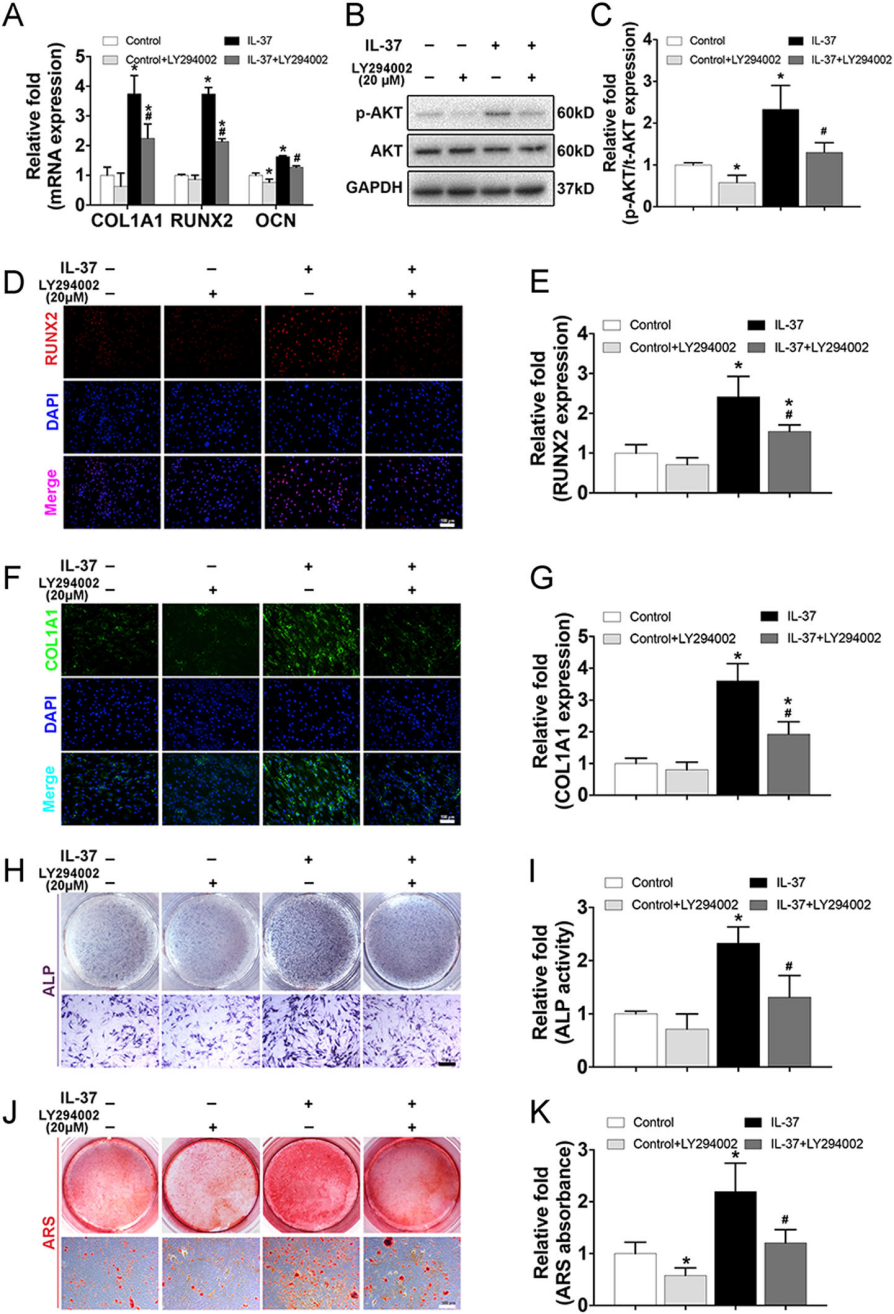
The enhanced osteogenic differentiation of MSCs due to the supplement of IL-37 was partially rescued by the presence of PI3K/AKT signaling inhibitor. **a** The increased mRNA levels of COL1A1, RUNX2, and OCN induced by IL-37 treatment (100 ng/ml) were significantly decreased following the addition of 20 μM LY294002 for 3 days; **b** Western blot analyses on day 3 of osteogenesis showed that the level of p-AKT was significantly decreased compared with the level in IL-37 (100 ng/ml) treated BMSCs without the inhibitor; **c** Relative quantitative analysis of western blot analyses; **d**, **e** Immunofluorescence staining of RUNX2 and its relative quantitative analysis, scale bar = 100 μm; **f**, **g** Immunofluorescence staining of COL1A1 and its relative quantitative analysis, scale bar = 100 μm; **h** Results of ALP staining, scale bar = 500 μm; **i** Results of relative ALP activity; **j**, **k** Results of ARS and its relative quantitative analysis, scale bar = 500 μm. *P < 0.05 vs. control group; ^#^*P* < 0.05 vs. IL-37 group

To verify the involvement of the PI3K/AKT signaling pathway, we examined the inhibitory effects of this pathway on osteogenesis by using an inhibitor of PI3K/AKT (LY294002 (20 μM))^30^. As is shown in Fig. 4a, the increased mRNA levels of COL1A1, RUNX2, and OCN induced by IL-37 treatment (100 ng/ml) were significantly decreased following the addition of LY294002 for 3 days. In addition, the level of p-AKT was also significantly decreased compared with the level in IL-37 (100 ng/ml) treated BMSCs without the inhibitor (Fig. 4b, c). Again, IF analysis confirmed the level changes of COL1A1 and RUNX2 (Fig. 4d–g). Moreover, inhibition of PI3K/AKT signaling pathway partially reversed the increase in osteogenesis of BMSCs, as indicated by ALP staining and ARS (Fig. 4h–k).

### IL-37 accelerated bone healing in a rat calvarial bone defects model

**Fig. 5:**
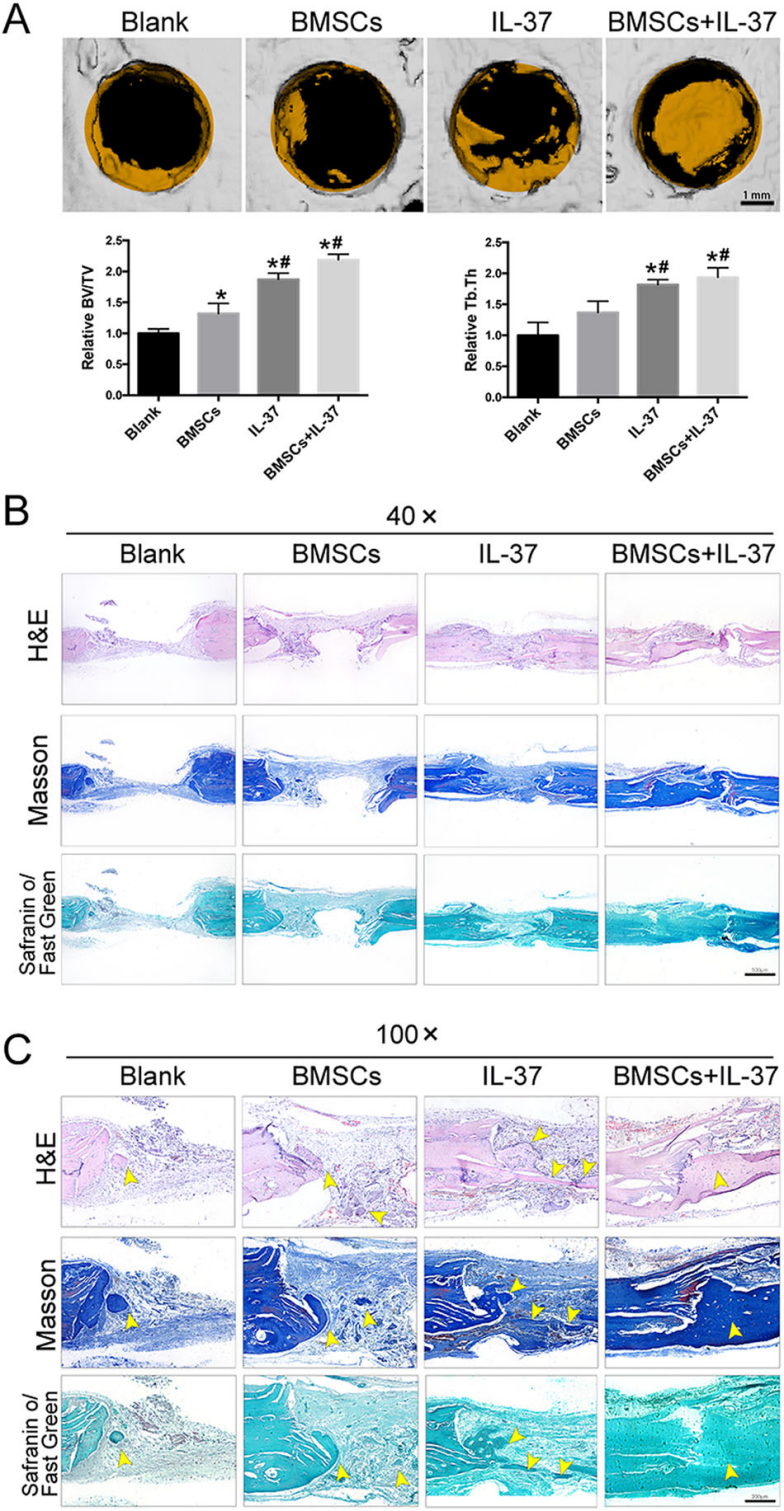
IL-37 accelerated bone healing in a rat calvarial bone defects model. **a** 3D construction images of μCT analyses and quantitive μCT analyses of BV/TV and Tb.Th, scale bar = 1 mm; **b**, **c** Histological analyses, including H&E, SO/FG, and Masson’s trichrome staining, showed that the calvarial defects in the Blank and BMSC groups were filled with fibrous tissue and a few bridging bone formation. In the IL-37 group, a thick callus consisting of newly formed bone tissue was observed in the defect area. In the BMSC+IL-37 group, large and thick callus was observed in the defect area and the remaining defect size was significantly smaller than other groups, indicating more complete bone healing of the defect, scale bars = 500 μm. Yellow arrowheads: the newly formed bone tissue. *P < 0.05 vs. Blank group; ^#^*P* < 0.05 vs. BMSCs group

To further evaluate the effect of IL-37 in vivo, human BMSCs with/without IL-37 were used in a rat calvarial bone defects model. The effect was confirmed by radiographic and histological analysis. The results of microcomputed tomographic (μCT) analyses showed a significant increase in trabecular bone volume per total volume (BV/TV) and trabecular thickness (Tb.Th) values in the IL-37 group and BMSC+IL-37 group when compared with the Blank group. Most importantly, the increase was significantly greater in the IL-37 group and BMSC+IL-37 group than the BMSC group. The results of the three-dimensional (3D) reconstruction of the μCT results showed that the largest defect appeared in the Blank group, while significant smaller defects were observed in the IL-37 group and BMSC+IL-37 group (Fig. 5a).

**Fig. 6:**
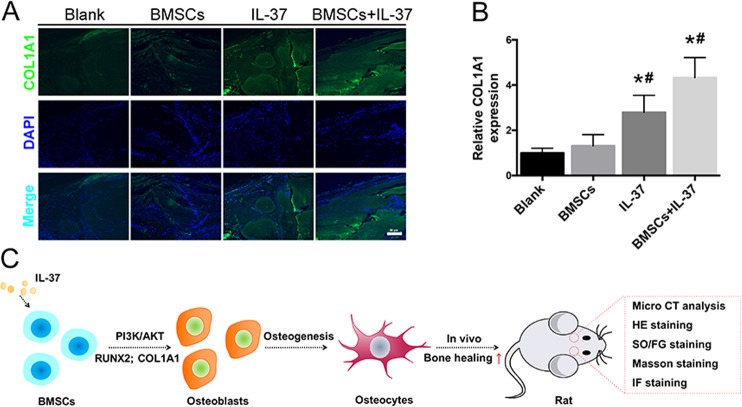
IL-37 increased the COL1A1 expression around the calvarial bone defect area. **a** Immunofluorescence staining showed that COL1A1 expression was increased in the IL-37 and BMSC+IL-37 groups; **b** Relative quantitative analysis of COL1A1 immunofluorescence staining; **c** The summary of design and findings of our study. Scale bar = 50 μm. *P < 0.05 vs. Blank group; ^**#**^*P* < 0.05 vs. BMSC group

Histological analyses including hematoxylin and eosin (H&E), Safranin O/Fast Green (SO/FG), and Masson’s trichrome staining showed that the calvarial defects in the Blank and BMSC groups were filled with fibrous tissue and a few bridging bone formations. In the IL-37 group, a thick callus consisting of newly formed bone tissue was observed in the defect area. In the BMSC+IL-37 group, large and thick callus was observed in the defect area and the remaining defect size was significantly smaller than the other groups, indicating more complete bone healing of the defect (Fig. 5b, c). In addition, using IF staining, COL1A1 expression was also found to be increased in the IL-37 group and BMSC+IL-37 group (Fig. 6a, b).

## Discussion

In the present study, we showed that IL-37, a new member of the IL-1 family that plays important roles in innate immunity and inflammatory responses in tumors and autoimmune diseases^20,25,31–35^, promoted the osteogenic differentiation of BMSCs. We found that extracellular IL-37 accelerated osteogenesis of BMSCs via activation of the PI3K/AKT signaling pathway in vitro. Moreover, local injection of IL-37 accelerated healing in a rat calvarial bone defect model. These findings indicated that IL-37 enhanced osteogenesis of BMSCs, at least in part by activation of the PI3K/AKT signaling pathway (Fig. 6c).

Previous data have shown that IL-37 is a pivotal anti-inflammatory cytokine and a fundamental inhibitor of innate immunity. Inhibiting the synthesis of IL-37 protein using small interfering RNA in PBMCs increased the production of pro-inflammatory mediators, including IL-1, IL-6, IL-12, TNF, and granulocyte macrophages colony-stimulating factor^32^. In another clinical study, rhIL-37 significantly downregulated the expression of TNF-α, IL-17, and IL-6 in PBMCs obtained from RA patients, whereas anti-TNF-α therapy significantly decreased the serum levels of IL-37^36^. A protective role of IL-37 in human malignancies, including fibrosarcoma, cervical cancer, hepatocellular carcinoma, breast cancer, and non-small-cell lung cancer, via the regulation of signaling pathways has also been reported, e.g., suppression of nuclear factor-κB and MAPK and activation of the Mer–PTEN-DOK pathway^37^. IL-37 has also been reported to protect against obesity-induced inflammation and insulin resistance^35^, as well as spinal cord injury^38^, although it remains unclear whether IL-37 is involved in osteogenesis or bone formation. This study emphasized a novel and promising role of IL-37 in regulating the osteogenic differentiation of BMSCs.

The concentrations of IL-37 used in this study were based on previous studies as well as normal serum levels of IL-37 in humans, which have been reported to be approximately 200 pg/ml in healthy individuals^22^. We used CCK-8 to assess the effects of 0–100 ng/ml IL-37 on the proliferation of BMSCs (Fig. 1a). Thus far, two studies regarding a relationship between IL-37 and OP have been reported^22,24^. AS patients with OP were reported to have significantly higher serum IL-37 levels than those without OP [239.970 (386.146–107.470) vs. 193.888 (93.536–294.802), *P* < 0.05]^22^. The increased serum IL-37 levels in OP patients, as we know, may due to the feedback response of OP development. As no significant difference in serum IL-37 levels was found between AS patients and healthy individuals, this demonstrated a potential link between IL-37 levels and bone metabolism. To some extent, these previous studies further supported our findings that IL-37 plays an important role in osteogenesis.

Bone healing occurs in four main phases: the early inflammatory phase, soft callus formation, hard callus formation, and bone remodeling; these have been described in detail in our previous study^39^. Briefly, when healing of the bone defect begins, the hematoma around the bone defect site is rich in inflammatory molecules, including IL-1β, IL-6, IL-8, TNF-α, IL-10, and high mobility group box 1 (HMGB1). In addition to many molecules, including IL-1β, IL-6, IL-8, and TNF-α, that have been widely investigated for their effects on MSC osteogenic differentiation, we have reported the roles of IL-10 and HMGB1 in osteogenesis^40–42^. Considering that IL-37 is constitutively expressed not in normal tissues but in tissues under inflammatory conditions, consistent with the finding that IL-37 mediates a negative feedback mechanism to curb inflammatory damage^32^, we believe that the in vivo positive effect of IL-37 on bone healing may be due not only to enhanced osteogenic differentiation of MSCs but also to its regulation of the inflammatory microenvironment surrounding the bone defect.

The PI3K/AKT signaling pathway has been shown to be critical for all phases of osteoblast differentiation, maturation, and bone growth^30,43–45^. Blocking the PI3K/AKT signaling pathway not only impairs chondrocyte differentiation but also inhibits longitudinal bone growth^46^. Moreover, the IL-37–PI3K/AKT axis is critically involved in the immune system. IL-37 was found to be a novel pro-angiogenic factor in developmental and pathological angiogenesis through activation of the PI3K/AKT signaling pathway^33^. In a study by Zhu et al., the anti-allergic inflammatory activity of IL-37 was partly mediated by the PI3K/AKT signaling pathway^47^. Regulation of the PI3K/AKT pathway was also reported to be responsible for inducing autophagy in hepatocellular carcinoma cells in an IL-37-dependent manner^31^. In the present study, we showed that IL-37 activated the PI3K/AKT signaling pathway during osteogenesis. A specific inhibitor of the PI3K/AKT pathway was used to inhibit the phosphorylation of a targeted protein. RT-PCR and western blot analyses, ALP staining, and ARS and IF analyses further confirmed the regulatory role of the IL-37–PI3K/AKT axis in the osteogenic differentiation of BMSCs.

To the best of our knowledge, this is the first study to investigate the effects of IL-37 on MSC differentiation. However, some limitations should be noted. First, although we investigated the role of IL-37 in osteogenesis, the role of IL-37 in other important processes, including osteoclastogenesis and adipogenesis, remains to be uncovered in further studies. Second, it should also be noted that inflammation levels can significantly affect osteogenesis^15–18,48^. In this study, we studied the role of IL-37 in physiological osteogenesis process of BMSCs, whereas the relationship between inflammation and IL-37 remains unknown. Thus further studies focusing on the role of IL-37 in osteogenesis under pathological conditions, especially inflammatory environment, will be of great interest. Third, the variants of IL-37, together with its intracellular and extracellular functionality, were not studied, which may decrease the robustness of the conclusions of this study. Fourth, we did not apply LY294002 in the bone defect animal model. Future in vivo studies using LY294002 would be helpful to validate our results. Also, kinetic in vivo measurements at different time points should also be conducted in future studies. Nonetheless, this study involved both in vitro and in vivo experiments exploring the role of IL-37 in osteogenesis, providing insight into the molecular mechanism of bone defect healing and the potential effects of IL-37 in regulating the osteogenic differentiation of BMSCs. Thus further studies are needed.

## Conclusions

Based on the results of this study, we found that extracellular IL-37 enhanced osteogenesis of BMSCs, at least in part by activation of the PI3K/AKT signaling pathway. The findings of our study provide insight and a possible novel target for large bone defect or non-union therapy; however, further studies are needed.

## Materials and methods

### Cell culture and reagents

Human BMSCs were purchased from Cyagen Biosciences (Guangzhou, China), which have been confirmed to be able to differentiate into osteoblasts, chondrocytes, and adipocytes under specific inductive conditions. Adherent BMSCs were cultured in human BMSC growth medium (Cyagen Biosciences, Guangzhou, China) in an incubator at 37 °C with 5% CO_2_. Cells were trypsinized and passaged after reaching 80% confluence. In subsequent experiments, cells from passages 3 to 5 were used.

rhIL-37 was purchased from R&D Systems (Shanghai, China). LY294002 (20 μM)^49^, a PI3K/AKT signaling pathway inhibitor, was prepared by Sigma-Aldrich (Shanghai, China). CCK-8 was purchased from Dojindo (Kumamoto, Kyushu, Japan). Antibodies against RUNX2 (#12556), phosphorylated-AKT (p-AKT, #4060), total-AKT (t-AKT, #4685), phosphorylated-p38 (p-p38, #4511), total-p38 (#8690), β-catenin (#19807), and glyceraldehyde-3-phosphate dehydrogenase (GAPDH, #2118) were obtained from Cell Signaling Technology (CST, Shanghai, China). Antibody against COL1A1 (ab34710) was obtained from Abcam (Abcam, Shanghai, China). Fetal bovine serum (FBS) was purchased from Gibco (Gibco, Australia). ARS Kits were supplied by Cyagen Biosciences (Guangzhou, China). SO/FG Staining Kit was from Sigma-Aldrich (Shanghai, China). Masson’s Trichrome Staining Kit was obtained from Nanjing Jiancheng Bioengineering Institute (Nanjing, China). ALP Activity Kit was purchased from Beyotime (Shanghai, China). All primers were synthesized by Sangon Biotech (Shanghai, China).

### Cell viability assay

To assess the effects of IL-37 on the viability of human BMSCs, cells were seeded into a 96-well plate (5000/well) and were allowed to adhere for 24 h. After that, various concentrations of IL-37 (0–100 ng/ml) were added to each well, with 3 replicates. After 1, 3, and 7 days, the medium was removed, and the cells were treated with 10% CCK-8 according to the manufacturer’s instructions. Absorbance at 450 nm was measured using a microplate reader (ELX808; BioTek, Winooski, VT, USA).

### Osteogenic differentiation of BMSCs

For osteogenic differentiation, human BMSCs (3 × 10^4^/cm^2^) were cultured by osteogenic differentiation medium (ODM; Cyagen Biosciences), human BMSC osteogenic differentiation basal medium with 10% FBS, 100 nM dexamethasone, 0.05 mM L-ascorbic acid-2-phosphate, and 10 mM β-glycerophosphate. The cells were maintained by replacing fresh ODM every 3 days. In addition, IL-37 was added to the ODM at concentrations ranging from 0.01 to 100 ng/ml^20,33,34^.

### ALP staining and activity assay

For ARS, ALP staining, and activity assay, BMSCs were seeded into 12-well plates and treated with ODM and IL-37 at various concentrations. For ALP staining, the cells that have been treated with ODM for 7 days were fixed with 4% paraformaldehyde (Sigma) for 15 min. Then the cells were washed three times with phosphate-buffered saline (PBS) and stained with an ALP Staining Kit (Beyotime, Shanghai, China). For the measurement of ALP activity, an ALP Activity Assay Kit (Beyotime) was used and BMSCs that have been treated with ODM for 3 and 7 days were lysed with a lysis buffer consisting of 20 mM Tris–HCl (pH 7.5), 1% Triton X-100, and 150 mM NaCl and incubated at 37 °C for 30 min according to the manufacturer’s instructions. The ALP activity was determined at 405 nm using a microplate reader (ELX808; BioTek).

### Assessment of calcium deposition

After treating the BMSCs with ODM for 14 days, an ARS Kit (Cyagen, Guangzhou, China) was applied to assess the mineral deposition. In brief, cells were fixed with 4% paraformaldehyde (Sigma) for 15 min and then washed three times with distilled water. After that, cells were incubated with 0.5% solution of ARS solution for 30 min at room temperature, followed by rinsing with distilled water. To determine the relative value of ARS, the stain was then desorbed with 10% cetylpyridinium chloride (MilliporeSigma, Billerica, MA, USA) for 1 h. After that, 200 μl aliquots of the solution were collected and plated on 96-well plates, which were read at 560 nm using a microplate reader (ELX808; BioTek). The readings of all samples were normalized to the total protein concentration.

### IF analysis of cells

Cells (3 × 10^4^/cm^2^) were cultured in a 12-well plate, and RUNX2, COL1A1, and p-AKT were detected using a fluorescence microscope (EU5888; Leica, Wetzlar, Germany) on day 3 after the induction of osteogenesis. Briefly, BMSCs were fixed in 4% paraformaldehyde (Sigma) for 15 min at room temperature. Then cells were permeabilized for 30 min in 0.05% Triton X-100 and blocked with 5% bovine serum albumin (BSA) for another 30 min. Fixed cells were then washed 3 times with PBS and incubated at 4 °C overnight with anti-RUNX2 (1:1600; CST), COL1A1 (1:500; Abcam, Shanghai, China), or p-AKT (1:400; CST). Cells were then incubated with a fluorescence-conjugated secondary antibody (Beyotime) for 2 h at room temperature, and the nuclei were then stained with 4′,6-diamidino-2-phenylindole (KeyGen Biotech, Nanjing, China) for 4 min. The results of IF were then observed and recorded under a fluorescence microscope (Leica, Solms, Germany).

### RNA extraction and RT-PCR

Three and 7 days after the induction of osteogenesis, total RNA from BMSCs was isolated using RNAiso reagent (Takara, Dalin, China) and quantified by measuring the absorbance at 260 nm (NanoDrop 2000; Thermo Fisher Scientific, MA, USA). Complementary DNA (cDNA) was synthesized using total RNA (≤1 μg) in a reaction volume of 20 μl using a cDNA Synthesis Kit (Takara). RT-PCR was then performed using Power SYBR^®^ Green PCR Master Mix (Takara) on the ABI StepOnePlus System (Applied Biosystems, Warrington, UK) to quantify all gene transcripts. 18S was used as a housekeeping gene. The detailed primer sequences of all genes are shown in Table 1. The cycle threshold (2^−△△Ct^ method) was used to evaluate the relative expression levels of target genes.

**Table 1:** Sequences of primers for quantitative real-time PCR

Gene	Forward (5′–3′)	Reverse (3′–5′)
ALP	TTGACCTCCTCGGAAGACACTCTG	CGCCTGGTAGTTGTTGTGAGCATAG
RUNX2	ACTTCCTGTGCTCGGTGCT	GACGGTTATGGTCAAGGTGAA
COL1A1	GAGAGCATGACCGATGGATT	CCTTCTTGAGGTTGCCAGTC
OCN	TGAGAGCCCTCACACTCCTC	CGCCTGGGTCTCTTCACTAC
OPN	CTCCATTGACTCGAACGACTC	CAGGTCTGCGAAACTTCTTAGAT
18S	CGCCGCTAGAGGTGAAATTC	TTGGCAAATGCTTTCGCTC

### Western blot analysis

To determine the protein expression of certain markers, protein extracts from BMSCs (day 3 or day 7 after the induction of osteogenesis) were prepared in radioimmunoprecipitation assay lysis buffer (Beyotime) supplemented with a proteasome inhibitor (Beyotime). Total proteins were separated by 10% sodium dodecyl sulfate-polyacrylamide gel electrophoresis and then transferred to a polyvinylidene difluoride membrane (Millipore, Shanghai, China). The membranes were then blocked in 5% BSA at room temperature for 1 h and then incubated overnight at 4 °C with antibodies specific to GAPDH (1:2000, CST), RUNX2 (1:1000; CST), COL1A1 (1:1000; Abcam), t-AKT (1:1000; CST), p-AKT (1:1000; CST), t-p38 (1:1000; CST), p-p38 (1:1000; CST), or β-catenin (1:1000; CST). After that, a secondary antibody (1:5000, Boster Biologic Technology, Wuhan, China) was applied for 2 h at room temperature. The immunoreactive bands were finally visualized and quantified using a Bio-Rad XRS chemiluminescence detection system (Bio-Rad, CA, USA).

### In vivo experiments

All animal experiments in this study were approved by the Institutional Animal Care and Use Committee of the Second Affiliated Hospital, School of Medicine, Zhejiang University, which were performed following the Animal Care and Use Committee guidelines of Zhejiang province together with the laboratory animals’ care and use guidelines.

In total, 20 male Sprague Dawley rats (8-week-old, weighing 250–300 g) provided by the Academy of Medical Sciences of Zhejiang Province were used to establish a rat calvarial bone defects. The rats were divided evenly and randomly into four groups (*n* = 5 per group): (1) Blank group: defects left untreated; (2) BMSC group: defects treated with BMSC sheets; (3) IL-37 group: defects treated with local injection of rhIL-37 (2 μg IL-37 in 200 μl normal saline); and (4) BMSC+IL-37 group: defects treated with BMSC sheets together with local injection of rhIL-37 (2 μg IL-37 in 200 μl normal saline). The details of BMSC sheet preparation and implantation were consistent with our previous studies^39,42,50,51^. In brief, confluent human BMSCs (1 × 10^5^/cm^2^) were cultured in flasks using MSC growth medium supplemented with vitamin C (20 µg/ml) for 2 weeks. After the formation of a sheet of BMSCs, cells were detached from the substratum as a cell sheet using a scraper.

The calvarial defect model was established as reported previously^52–54^. In brief, 0.3% pentobarbital sodium (Sigma) at 30 mg/kg body weight was used intraperitoneally to induce anesthesia. After that, a 1.5-cm incision in the sagittal direction was made to expose the cranium. Then a 4-mm-diameter defect was made using a low-speed dental engine with a burr on both sides of the cranium. The incision was then closed with 4–0 absorbable sutures. Referring to the methods in former in vivo studies^25,55,56^, 2 μg IL-37 in 200 μl normal saline were locally injected in the calvarial defect sites of the rats from IL-37 group and BMSC+IL-37 group every 2 weeks after surgery, while rats from the Blank group and BMSC group were treated with same volume normal saline. Eight weeks after surgery, all the rats were sacrificed in a CO_2_ chamber. The cranial specimens were collected and fixed in 4% paraformaldehyde (Sigma) for 48 h at room temperature and kept in PBS for radiographic and histological analyses.

### μCT scanning

To evaluate the bone formation at the calvarial defect sites, cranium samples (*n* = 5 for each group) were scanned at 8 weeks after the surgery using a high-resolution μCT-100 imaging system (Scanco Medical, Brüttisellen, Switzerland) with X-ray energy settings of 70 kV and 80 μA, 14.8 μm thickness with an exposure time of 300 ms. After 3D reconstruction, a square region of interest was selected to conduct bone morphometric analysis including BV/TV and Tb.Th^39,50,51,57^.

### Histological evaluation

After μCT scanning, specimens from rats (*n* = 5 for each group) were then decalcified in 10% ethylene diamine tetraacetic acid (Sigma) with 0.1 M PBS for >2 months, with a solution change every 3 days. Thereafter, the decalcified specimens were embedded in paraffin using standard procedures. Serial sections (4 µm thickness) were stained with H&E, SO/FG, and Masson’s trichrome separately in accordance with our previous studies^39,50,51,57^. In addition, IF staining of COL1A1 was also applied using standard methods^58^. Images were obtained on a microscope (Leica, Solms, Germany).

### Statistical analysis

Statistical analysis was applied using the SPSS 19.0 software (IBM, NY, USA). All experiments were performed at least in triplicate, and the data were all presented as means ± standard deviation (SD). Statistical differences were assessed using one-way analysis of variance followed by Bonferroni’s post hoc test. *P* value ≤0.05 was considered statistically significant.
